# Attentional vigilance of food information in disordered eating behaviors

**DOI:** 10.3389/fpsyt.2023.1108995

**Published:** 2023-02-15

**Authors:** Luyao Jin, Wenyue Han, Zheng Zheng

**Affiliations:** ^1^School of Medicine & Holistic Integrative Medicine, Nanjing University of Chinese Medicine, Nanjing, China; ^2^Changzhou Vocational Institute of Textile and Garment, Changzhou, China

**Keywords:** disordered eating behavior, attentional bias, exogenous cueing task, attentional vigilance, female college students

## Abstract

**Introduction:**

Disordered eating behaviors (DEBs) are very common among female college students, which seriously endanger their health and well-being. Therefore, the study of the mechanism of DEBs can provide effective evidence for early detection and intervention.

**Methods:**

In total of 54 female college students were recruited and assigned to DEB group (*n* = 29) and healthy control (HC) group (*n* = 25) according to their scores in the Eating Attitudes Test-26 (EAT-26). Then, the Exogenous Cueing Task (ECT) was used to evaluate their reaction time (RT) to the location of a target dot preceded by a food or neutral cue.

**Results:**

The study found that compared with HC group, DEB group showed more attentional engagement to food stimuli, indicating that attentional vigilance to food information could be considered as a specific attentional bias of DEBs.

**Discussion:**

Our findings not only provide evidence of the potential mechanism of DEBs from the perspective of attentional bias, but also can be considered as an effective and objective indicator for early screening of subclinical eating disorders (EDs).

## 1. Introduction

The term disordered eating behaviors (DEBs) includes a wide spectrum of eating pathologies, such as strict eating, binge eating, fasting, emotional eating, and out-of-control eating, at a frequency or severity that does not meet the criteria for an eating disorder (ED) (1–5). DEBs are prevalent among adolescents (6, 7), and have significant gender difference, with females higher than males (7–9). The incidence rate of DEBs among the population aged 11–18 years in Greece and the United States was 12–18% (10, 11), and that of female college students in the United States was 11–20% (2).

DEBs seriously damage the individual’s psychosomatic health, for example, affect self-evaluation (1), impair academic achievement (12), cause alexithymia (9). DEBs often comorbid with other mental disorders, such as anxiety (10), depression (13), substance abuse (14), and personality disorder (15), and are highly predictive of suicidal tendencies (16). More importantly, DEBs will greatly increase the risk of developing into EDs in the future, such as binge eating and anorexia nervosa (5, 17). DEBs in adolescence strongly predict ED symptoms after 5 years (18). Therefore, DEBs have a significant negative impact on personal health and wellbeing and social care costs. Thus, a better understanding of the potential mechanism of DEBs can not only alleviate the physical and mental pain of individuals, but also reduce the incidence of EDs.

Attentional bias (AB) refers to an individual’s attention allocation characteristics to threat stimulus relative to neutral stimulus (19). Patients with EDs show attentional bias (AB) toward food-related stimuli (20–22). In the exogenous cueing task (ECT) paradigm, food pictures with emotional potency are defined as threat-related stimuli (23). Using ECT, researchers found that compared with control group, women with binge eating showed difficulty in separating attention from high calorie foods (24), while anorexia nervosa like patients showed attentional avoidance of high-fat foods (25). Since patients with EDs show a clear AB to food stimuli, do DEBs also exhibit specific AB to food stimuli?

Although some previous studies have found that DEBs are related to attentional bias, there is no consistent evidence so far. For example, Veenstra et al. (23) found through the ECT that both restrained and unrestrained eaters showed avoidance of high-fat foods, but did not avoid low-fat foods. Meule et al. (26) found through the Flanker task that compared with the neutral pictures, the response time of restricted eaters to high-calorie food cues was faster than that of unrestrained eaters. Through the Stroop Task, Hodge et al. (27) did not find that non-clinical female restrained eaters had attentional bias to food words. Brignell et al. (28) and Hou et al. (29) found through the pictorial visual-probe task that individuals with high-external eating showed an enhanced attentional bias for pictorial food cues. Therefore, in order to clarify the specific AB of DEBs to food-related stimuli, the present study used ECT to compare the attentional characteristics of female college students in DEB group and healthy control group.

## 2. Materials and methods

### 2.1. Participants

**FIGURE 1:**
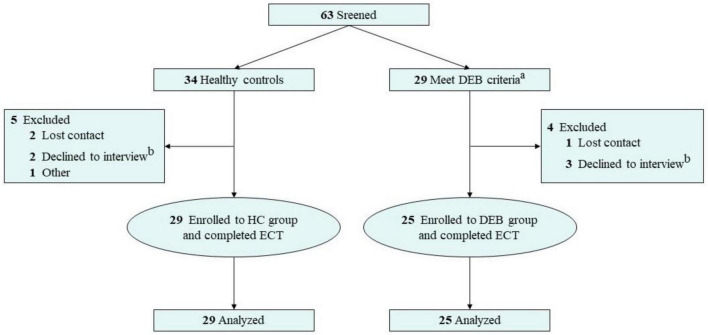
Process of recruitment. DEB, disordered eating behavior; HC, healthy control; ECT, exogenous cuing task. ^a^The Eating Attitudes Test-26 score greater than 10 is considered to have DEBs. ^b^Specific content of the interview: (1) Understand the subjects’ eating status; (2) Find out whether the subjects meet the inclusion and exclusion criteria; (3) Introduce the research purpose, experimental process, subjects’ rights and obligations, etc.; (4) If the subjects agree to join the group, sign informed consent. (5) Complete ECT.

This study was conducted in five universities in Nanjing, China, from May 2021 to October 2022. Some of the subjects were students who participated in psychological class and were invited to participate in the questionnaire voluntarily, while others were recruited through posters. Social media was also used to invite potential candidates. The following contents were used in the recruitment poster: “Do you often pay attention to eating? Do you have emotional eating, dieting, overeating or other disordered eating behaviors? Do you want to improve your eating behaviors through psychological methods?” We received 78 willingness to participate, of which 63 (80.8%) were women and 15 (19.2%) were men. This result was in line with the demographic characteristics of DEBs. In order to ensure the homogeneity of the research objects, female college students were selected as participants. Finally, 54 female college students completed the experiment, including 29 in HC group and 25 in DEB group. They provided written informed consent before the start of the study, and each person was paid 100 RMB after the experiment. The screening process of participants is shown in Figure 1.

The inclusion criteria included: (a) female college students who volunteered, (b) receiving the measurement of Eating Attitude Test-26 (EAT-26) (score less than or equal to 10 was classified as HC group and score more than 10 was classified as DEB group), (c) receiving interview based on structured clinical interview for DSM-V (SCID) to exclude EDs, and (d) the body mass index (BMI) was between 15 and 27.

The exclusion criteria included: (a) the existence of psychological disorders, such as EDs, schizophrenia, depression, anxiety disorder, personality disorder, etc. (b) the existence of organic diseases, and (c) having received or undergoing psychological or drug treatment related to eating problems.

### 2.2. Measures

Eating Attitudes Test-26 (EAT-26) is a self-report questionnaire containing 26 items proposed by Garner et al. (30). Each item is scored using six points, from 1 (never) to 6 (always). EAT-26 is divided into three dimensions: dieting (e.g., I avoid eating even if I am hungry), bulimia and food preoccupation (e.g., I often think about food), and oral control (e.g., I eat slowly). The reliability of the total scale is 0.9. Combined with previous studies on EAT-26 and eating behavior characteristics of Chinese people, in this study, we classified less than or equal to 10 points into the HC group and more than 10 points into the DEB group (31–33). Internal consistency in the present study was 0.78.

### 2.3. Experimental tasks

#### 2.3.1. Stimulus

##### 2.3.1.1. Food stimulus

According to the eating habits of Chinese young people, food pictures were selected from Roefs et al. (34) and Veenstra et al. (23) study and the Internet. Fifty female college students (randomly selected in the psychological class and did not participate in the follow-up experiment) were asked to evaluate these pictures from three dimensions: (a) subjective evaluation of calories (nine-point scores, one indicated low calories and nine indicated high calories); (b) pleasure (nine-point scores, one indicated low pleasure and nine indicated high pleasure); (c) arousal (nine-point scores, one indicated low arousal and nine indicated high arousal). According to the total score of these three dimensions of each food picture, the top 35 food pictures from high to low were selected (M ± SD: calorie = 6.11 ± 1.02, pleasure = 5.21 ± 0.90, arousal = 5.62 ± 1.15). From the 35 pictures, 30 pictures were randomly selected to form a food picture database, and the remaining five pictures were used as exercise pictures. Finally, 20 food pictures were randomly selected from the food picture database for the formal experiment (see Supplementary material).

##### 2.3.1.2. Neutral stimulus

Seventy neutral pictures of daily necessities were selected from International Affective Picture System (IAPS) and the Internet. Fifty female college students (randomly selected in the psychological class and did not participate in the follow-up experiment) were asked to evaluate these pictures from two dimensions: (a) pleasure (nine-point scores, 1–4 points indicated negative emotions, such as anger, fear, safety, etc., five points indicated neutral emotion, and 6–9 points indicated positive emotions, such as happiness, etc.); (b) arousal (ine-point scores, 1–4 points indicated no obvious emotional ambient, such as feeling calm, relaxed and not alert, five points represented general emotion, 6–9 points indicated obvious emotion, such as extremely excited, stimulated, excited or angry and excluded). According to the total score of the two dimensions of each neutral picture, the middle 35 pictures were selected from high score to low score (M ± SD: pleasure = 4.08 ± 0.90, arousal = 3.82 ± 1.14). From the 35 pictures, 30 pictures were randomly selected to form a neutral picture database, and the remaining five pictures were used as exercise pictures. Finally, 20 neutral pictures were randomly selected from the neutral picture database for the formal experiment (see Supplementary material).

#### 2.3.2. Exogenous cueing task

The exogenous cueing task (ECT) was first proposed by Posner and Cohen (35) and then improved by Koster et al. (36) to measure subject’s AB to specific stimulus. Reaction times (RTs) were collected with E-prime 2.0, and all stimuli were presented on the computer screen.

**FIGURE 2:**
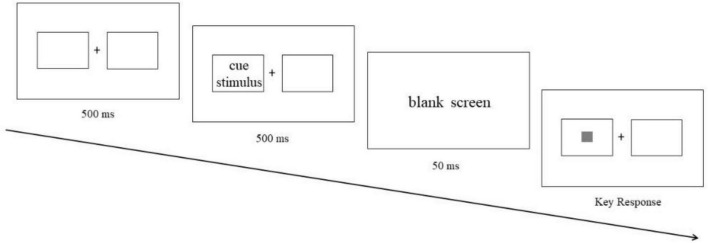
An example of valid trial in exogenous cuing task (ECT).

**FIGURE 3:**
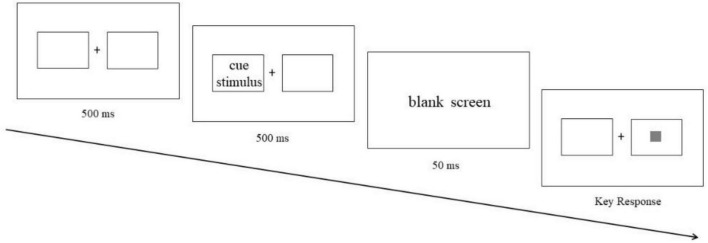
An example of invalid trial in exogenous cuing task (ECT).

An ECT trial started with a 500 ms fixation point “+” and two boxes on both sides in the center of the screen. Then, cue stimulus (food picture or neutral picture) would appear in the box on the left or right, and the presentation time was 500 ms. After the cue stimulus disappeared, a 50 ms blank screen was presented (23). Finally, the target stimulus (gray box) would appear in the box on the left or right. Participants had to press the “A” key for the left target and the “L” key for the right target with their index fingers as soon as possible when the box appeared. If the target stimulus and cue stimulus appeared on the same side, it was a valid trial (see Figure 2); If the target stimulus and cue stimulus appeared on the opposite side, it was an invalid trial (see Figure 3). Valid trials were used to calculate attentional engagement, and invalid trials were used to calculate attentional disengagement (see “section 2.4 Date analysis” for details). The subjects completed 20 practice trials before 160 trials that were included in the formal experiment. The pictures used in the exercise were all exercise pictures that would not be used in the formal experiment. Each picture was presented four times (two valid trials: left stimulus-left target and right stimulus-right target; two invalid trials: left stimulus-right target and right stimulus-left target). Different trials were carried out randomly.

### 2.4. Data analysis

SPSS 22.0 was used for statistical analysis. Measurement data were expressed as mean ± standard deviation (M ± SD), and count data were expressed as number of cases (percentage). Differences in group characteristics were assessed using the Chi-square test and independent *t*-tests two-tailed with a significance level of 0.05. The analysis of ECT results was based on the average RT of the correct response to the target under different experimental conditions, that is, press the “A” key for the left target and the “L” key for the right target. The correct response with RT less than 200 ms or more than 750 ms was excluded because they might be caused by continuous keystrokes, distractions, etc. (36).

A 2 × 2 × 2 (Group × Cue Stimulus × Cue Validity) mixed-factor ANOVA was conducted, with the between-subjects factor of Group (DEB, HC) and the within-subject factor of Cue Stimulus (food, neutral picture), and Cue Validity (valid, invalid). Then, another set of 2 × 2 (Group × Cue Stimulus) mixed-factor ANOVA was conducted to explore whether subjects show attentional bias to food pictures vs. neutral pictures. The confidence interval percentage was 95%, and *p* < 0.05 was considered statistically significant.

Following indices of AB were calculated (25, 37, 38):

(1) Attentional engagement = RT *valid neutral cue*—RT *valid food cue*. A positive score indicates that attention is easier directed at the location of the food cue as compared to the neutral cue. A negative score indicates decreased attentional engagement with the food cue.

(2) Attentional disengagement = RT *invalid food cue*—RT *invalid neutral cue*. A positive score indicates slower disengagement of attention and thus a reduced ability to shift attention away from the food as compared to the neutral cue, difficulty in disengagement toward food cue stimulus. A negative score indicates faster disengagement of attention from the food cue.

## 3. Results

### 3.1. Participants disposition and demographics

Participants’ characteristics are shown in Table 1.

**TABLE 1:** Demographic characteristics of participants.

	DEB group (*n* = 25)	HC group (*n* = 29)	Test	*P*-value
Characteristic	M ± SD	M ± SD	–	–
Age (years)	20.52 ± 1.57	20.72 ± 1.37	t (52) = 5.10	0.61
Major (1:2:3)	12:8:5	11:10:8	X^2^ (2, *N* = 54) = 0.67	0.72
BMI	20.99 ± 2.51	19.87 ± 1.98	t (52) = 1.82	0.07
EAT-26	19.64 ± 6.71	5.45 ± 2.75	t (52) = 10.43	0.000
(1) Dieting	13.20 ± 4.64	3.14 ± 1.98	t (52) = 10.63	0.000
(2) Bulimia and food preoccupation	3.72 ± 3.97	0.59 ± 0.73	t (52) = 4.17	0.000
(3) Oral control	2.72 ± 1.57	1.79 ± 1.78	t (52) = 2.02	0.049

BMI, body mass index (calculated as weight in kilograms divided by height in meters squared); EAT-26, Eating Attitudes Test-26; DEB, disordered eating behavior; HC, healthy control. Major was defined on a three scale (1 = Medicine, 2 = Liberal arts, 3 = Science and engineering).

### 3.2. RT of different groups in ECT

#### 3.2.1. Overall effects

Table 2 displays the average RT in each group under different experimental conditions.

**TABLE 2:** Average reaction time (RT) (milliseconds) under different experimental conditions (M ± SD).

	Valid trial		Invalid trial	
	**Food cue stimulus**	**Neutral cue stimulus**	**Food cue stimulus**	**Neutral cue stimulus**
DEB group	397.09 ± 124.55	402.71 ± 125.72	388.93 ± 126.04	386.92 ± 123.64
HC group	402.27 ± 111.37	397.99 ± 105.91	389.91 ± 104.76	385.38 ± 101.34

DEB, disordered eating behavior; HC, healthy control.

The results of two (Group: DEB, HC group) × 2 (Cue Validity: valid, invalid cue) × 2 (Cue Stimulus: food, neutral picture) mixed analysis of variance (ANOVA) showed that the main effect of cue stimulus was not significant, F (1, 52) = 1.24, *p* = 0.27, the main effect of group was also not significant, F (1, 52) = 0.00, *p* = 1.00, the main effect of cue validity was significant, F (1, 52) = 11.45, *p* < 0.05, $\eta_{p}^{2}$ = 0.18, and RT under valid cue conditions (M = 400.02 ms, SD = 114.94 ms) was slower than that under invalid cue conditions (M = 387.79 ms, SD = 112.05 ms). The interaction between these three variables was not significant, F (1, 52) = 1.91, *p* = 0.17, and then attentional engagement and disengagement were further analyzed.

#### 3.2.2. Attentional engagement and disengagement

A 2 × 2 (Group × Cue Stimulus) mixed-factor ANOVA was conducted, with the between-subjects factor of Group (DEB, HC) and the within-subject factor of Cue Stimulus (food, neutral picture), to test the attentional engagement and disengagement.

**FIGURE 4:**
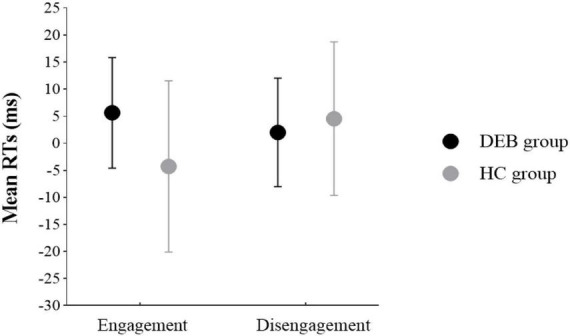
Attentional bias indices engagement and disengagement (in ms) for the disordered eating behavior (DEB) group and healthy control (HC) group. Vertical bars denote standard errors. Engagement: Mean RTs = RT valid neutral cue–RT valid food cue; Disengagement: Mean RTs = RT invalid food cue–RT invalid neutral cue.

The analysis of attentional engagement revealed a significant Group × Cue Stimulus interaction, F (1, 52) = 7.20, *p* < 0.05, $\eta_{p}^{2}$ = 0.12. The Bonferroni simple effect test showed that for DEB group, the RT to neutral pictures (M = 402.71 ms, SD = 125.72 ms) was slower than that to food pictures (M = 397.09 ms, SD = 124.55 ms), F (1, 52) = 4.31, *p* < 0.05, $\eta_{p}^{2}$ = 0.08, indicating that the subjects’ attention tended to point to the location of food cues. For HC group, there was no difference in RT to food pictures (M = 402.27 ms, SD = 111.37 ms) or neutral pictures (M = 397.99 ms, SD = 105.91 ms), F (1, 52) = 2.91, *p* = 0.94. The analysis of attentional disengagement revealed that the interaction between Group and Cue Stimulus was not significant, F (1, 52) = 0.55, *p* = 0.46. The results of attentional engagement and disengagement are shown in Figure 4.

## 4. Discussion

The purpose of this study was to examine whether female college students with DEBs had different attentional characteristics to food stimuli compared with HC group. The ECT was used to measure attentional engagement and attentional disengagement. The main finding of this study was that compared with HC group, DEB group showed more attentional engagement to food information, namely attentional vigilance, which could be considered as a specific AB of DEBs.

In our study, individuals with DEBs showed stronger attentional engagement than healthy controls, that is, they could be attracted to food stimuli faster and easier. This was consistent with the results of ECT and eye-tracking measurement in patients with binge eating disorder (39–41). Individual attention is attracted to certain stimuli more easily or faster, which is an early attentional vigilance (42). This early attentional vigilance is the ability to increase response readiness for a short period of time after external cues or stimuli (phasic alertness) (43). In our study, food pictures with high arousal and high pleasure might successfully trigger the threat detection mechanism of subjects with DEBs, which is the basis of attentional vigilance (42). The possible reason is that individuals with DEBs regard food stimulus as a signal of impending DEBs and psychological distress. Early attentional vigilance is a bottom-up cognitive processing of sensory information, which means that cognitive processing ability is automatically allocated to important stimuli (44). This further indicates that individuals with DEBs are extremely sensitive and quick to respond to threatening food signals, and have developed into automatic attention to food, which is an indicator of impulsive eating (45). Although the manifestations of DEBs are very diverse, including dieting, overeating, emotional eating, etc. (4, 5), eating impulse may be one of the core characteristics.

Our study also found that there was no difference between DEB Group and HC group in attentional disengagement from food stimuli, and neither group showed difficulty in attentional disengagement. One possible reason is that the difficulty in attentional disengagement is the combination of automatic processing and strategic processing (44). Attentional disengagement from food cues is considered a more top-down controlled process (46). When being automatically attracted by food stimuli, both groups of subjects simultaneously adopted cognitive reappraisal strategy, which indicated that there was no difference in their attention control. Another explanation is that the difficulty in attentional disengagement is related to food deprivation (47).

The innovation of this study is that we explored the attentional bias of DEBs to food information, which not only provides us with evidence of the potential mechanism of DEBs from the perspective of attentional bias, but also can be regarded as an effective and objective indicator for early screening of subclinical EDs. Of course, this study also has some limitations. In ECT, we only measured the RT when the cue was presented for 500 ms, but did not measure the effect of other presentation times on RT. Some studies have shown that longer stimulus presentation time (≥3,000 ms) may be more likely to produce reliable attentional bias (48). Therefore, future studies can compare the effects of different presentation times on attentional bias. The subjects in this study had multiple DEBs on the same person. Therefore, in future studies, subjects who only show a specific type of DEBs, such as restricted diet, overeating or emotional diet, can be selected to study the attentional bias characteristics of different types of DEBs. In addition, the subjects of this study were women, and men can be studied in the future.

## Data availability statement

The original contributions presented in this study are included in this article/Supplementary material, further inquiries can be directed to the corresponding author.

## Ethics statement

The studies involving human participants were reviewed and approved by the Ethics Committee of The Affiliated Hospital of Nanjing University of Chinese Medicine. The participants provided their written informed consent to participate in this study.

## Author contributions

LJ was responsible for the experiments, data analysis, and writing. WH was responsible for the recruitment of subjects. ZZ was responsible for experimental design and writing. All authors contributed to the article and approved the submitted version.
