# Supplementary figures and images for: Attentional vigilance of food information in disordered eating behaviors
Source: Front Psychiatry. 2023 Feb 15;14:1108995. doi: 10.3389/fpsyt.2023.1108995 (PMC9974645; doi:10.3389/fpsyt.2023.1108995)

Example of food pictures

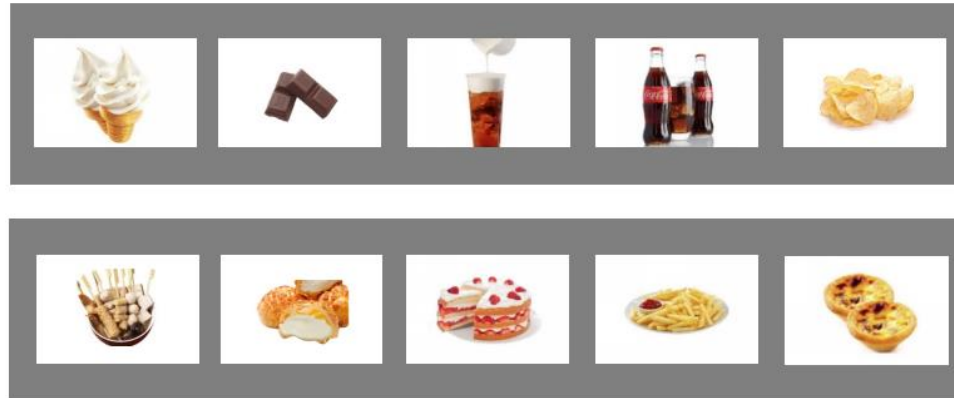

Example of neutral pictures

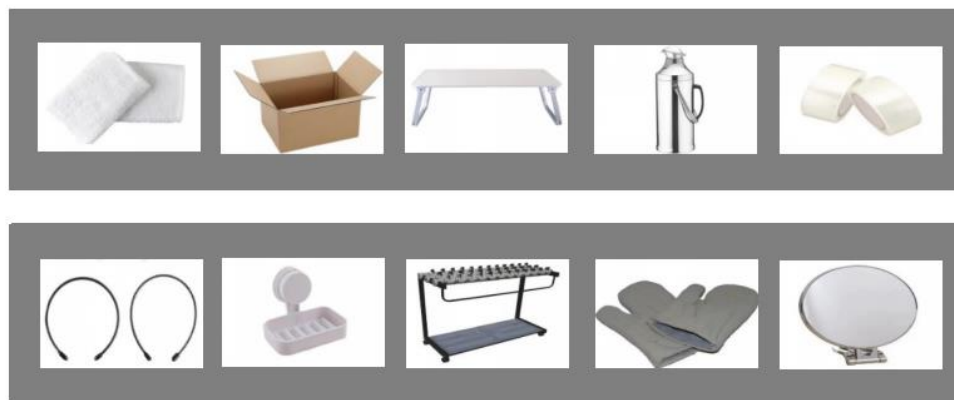

Supplement: Supplementary file 1 [file Data_Sheet_1.PDF]
